# Machine Learning Techniques, Applications, and Potential Future Opportunities in Pressure Injuries (Bedsores) Management: A Systematic Review

**DOI:** 10.3390/ijerph20010796

**Published:** 2023-01-01

**Authors:** Odai Y. Dweekat, Sarah S. Lam, Lindsay McGrath

**Affiliations:** 1 Department of Systems Science and Industrial Engineering, Binghamton University, Binghamton, NY 13902, USA; 2 Wound Ostomy Continence Nursing, ChristianaCare Health System, Newark, DE 19718, USA

**Keywords:** pressure ulcer, pressure injury, bedsores, machine learning, deep learning, systematic review, clinical decision support, hospital-acquired pressure injuries, predictive analytics, PRISMA, wound image analysis, artificial intelligence

## Abstract

Pressure Injuries (PI) are one of the most common health conditions in the United States. Most acute or long-term care patients are at risk of developing PI. Machine Learning (ML) has been utilized to manage patients with PI, in which one systematic review describes how ML is used in PI management in 32 studies. This research, different from the previous systematic review, summarizes the previous contributions of ML in PI from January 2007 to July 2022, categorizes the studies according to medical specialties, analyzes gaps, and identifies opportunities for future research directions. PRISMA guidelines were adopted using the four most common databases (PubMed, Web of Science, Scopus, and Science Direct) and other resources, which result in 90 eligible studies. The reviewed articles are divided into three categories based on PI time of occurrence: before occurrence (48%); at time of occurrence (16%); and after occurrence (36%). Each category is further broken down into sub-fields based on medical specialties, which result in sixteen specialties. Each specialty is analyzed in terms of methods, inputs, and outputs. The most relevant and potentially useful applications and methods in PI management are outlined and discussed. This includes deep learning techniques and hybrid models, integration of existing risk assessment tools with ML that leads to a partnership between provider assessment and patients’ Electronic Health Records (EHR).

## 1. Introduction

### 1.1. Pressure Injuries Formation

Skin damage incurred as a result of pressure injuries is painful, disfiguring, and costly to treat. The National Pressure Injury Advisory Panel (NPIAP) defines a pressure injury as localized damage to the skin and underlying soft tissue, usually over a bony prominence or related to a medical or other device [1]. Pressure Injuries are further categorized into two classifications: Hospital-Acquired Pressure Injuries (HAPI) and Community-Acquired Pressure Injuries (CAPI). The Centers for Medicare and Medicaid Services (CMS) consider HAPI to be “never events”. Hospital HAPI rates are therefore reported, and hospitals can be liable for financial penalties as well as reductions in hospital grades [1]. HAPI prevention has become a national focus, but identification of at-risk patients and personalizing care can be expensive, labor intensive, and potentially burdensome, both for the patient and the care providers. 

HAPI is one of the most common yet preventable health conditions in the United States and costs in excess of $ 26.8 billion annually [2]. Known by many names, such as Pressure Injuries (PI), bedsores, pressure ulcers, or decubitus ulcers, the injury develops as a result of pressure or pressure in combination with shear and friction, which results in tissue deformation or ischemia. 

**Figure 1:**
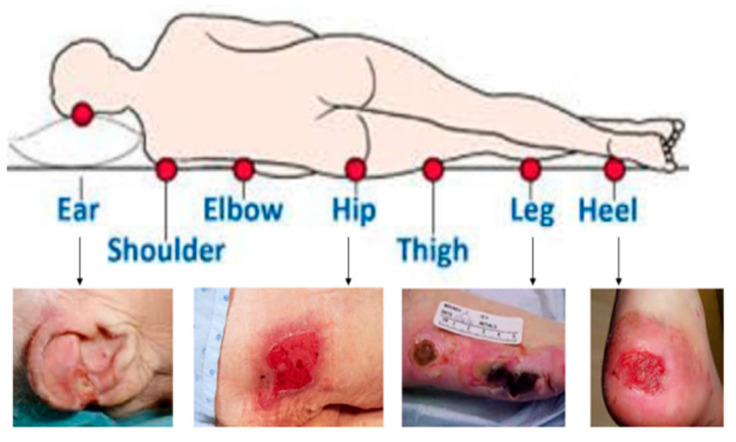
Different locations of PI [3].

HAPI can occur in almost any place on the body, although they tend to develop more frequently in locations where bony prominences are present or below areas where medical equipment is placed, as shown in Figure 1 [3]. 

### 1.2. Pressure Injuries Stages

**Figure 2:**
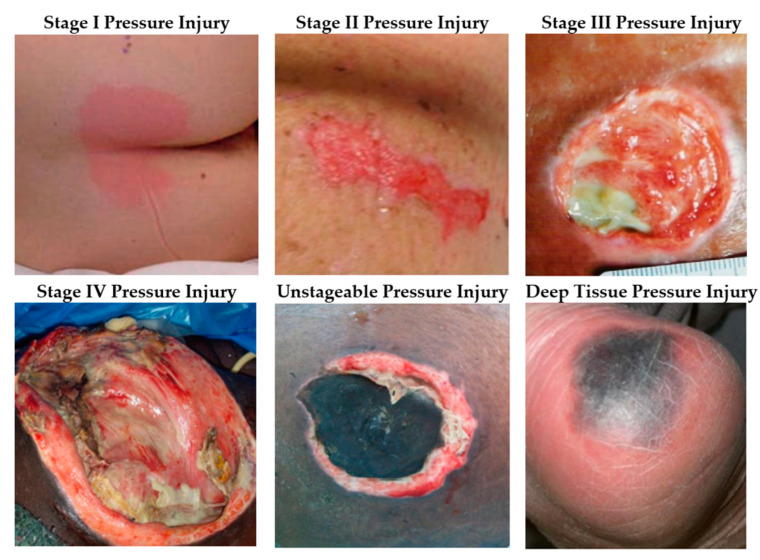
Stages of pressure injuries (adapted from [1]).

**Figure 3:**
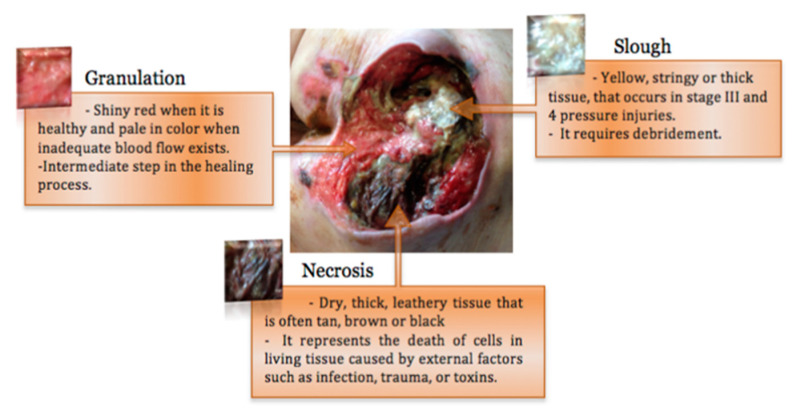
Main types of tissues in stage 3 and stage 4 PI [4].

Pressure injuries are graded into stages, according to the level of exposed tissue, as presented in Figure 2. Stage 1 pressure injuries present as intact skin with a localized area of non-blanchable (when light pressure is applied to close the capillary bed, then released the color does not change) erythema. Stage 2 presents as partial thickness skin loss with exposed dermis or the presence of a serum-filled blister. Stage 3 presents as full-thickness skin loss in which subcutaneous fat is visible. Stage 4, the most severe stage, presents as full-thickness skin and tissue loss, in which fascia, muscle, tendon, or bone exposed. The main types of tissues in stage 3 and stage 4 are adipose, granular, slough, and/or eschar; they are summarized in Figure 3 [4]. Unstageable injuries are advanced to where the extent of skin and tissue loss is obscured by slough or eschar. Deep Tissue Pressure Injuries (DTPI) are localized regions of non-blanchable, deep red, maroon, or purple discoloration, which may quickly develop as the extent of the wound is revealed [1]. 

### 1.3. Pressure Injuries Consequences 

Prevention of PI is a vital foundation for patient care and safety and a holistic treatment view needs to be adopted, while considering the patient’s tissue tolerance and condition, because HAPI can develop anywhere on the body that is subjected to pressure or pressure in combination with shear [5,6]. If HAPI develops, the injury will be staged based on the type of tissue noted in the wound bed. These injuries will lead to an increased length of hospital stay, increases resource requirements, and financial penalties, and if the injury expands further than the dermis, repeat damages to the area may lead to lifelong struggles for the patient. Early detection of at-risk patients as well as personalized HAPI care plans will undoubtedly reduce the incidence of HAPI. 

### 1.4. Pressure Injuries Prevention 

Through risk assessment and targeted prevention efforts, the majority of HAPI can be prevented; nevertheless, the majority of patients in acute care and long-term care settings are at risk, and the labor and product costs associated with prevention can be substantial. The most effective technique for handling HAPI is to prevent their occurrence. When an individual enters an inpatient setting, the first step is to employ a PI risk assessment tool [1]. 

Recognizing an individual’s risk of developing HAPI early enables the caregiver to implement prevention actions that lower the likelihood of increasing mechanical load upon the patient’s body and increase tissue tolerance. The earlier a patient’s risk is detected, the less likely it is that he or she would develop HAPI. Adopting a standardized risk assessment can assist in reducing the number of patients at risk for developing pressure injuries and reveal modifiable patient-specific risk factors. Additionally, it permits the caregiver to design a care plan and implement particular risk control strategies; however, many patients still remain at risk.

Over a hundred distinct risk variables have been identified as possible contributors to the development of pressure injuries. There are two sorts of factors: those that influence tissue tolerance and those that enhance mechanical load. These risk factors can be further divided into those that are modifiable and those that are not. Most hospitalized patients are at risk for developing pressure injuries due to the sheer number of risk variables; avoiding these injuries can be onerous in settings with limited resources and staffing levels [1].

### 1.5. Standardized Risk Assessment Tools and Risk Factors

Various PI risk assessment tools are available that categorize patients into risk groups. The most commonly used risk assessment tools include the Braden Scale, Waterlow Scale, Norton Scale, Cubbin-Jackson Scale, Spinal Cord Injury Pressure Ulcer Scale (SCIPUS), and Braden Q Scale [7,8,9,10,11,12,13,14,15]. The risk factors, advantages, disadvantages, and specialties of these risk assessment tools are summarized in Table 1.

The Norton Scale, for instance, can be used for broad applications. It consists of only five basic subscales: physical conditions, mental conditions, activity, mobility status, and incontinence/continence. However, Norton does not examine any of the other aspects covered by Braden, which include sensory perception, friction, skin moisture, and shear. In contrast, physical and mental conditions that are addressed by the Norton Scale are not addressed by the Braden Scale. Risk assessment encompasses a variety of modifiable risk factors. However, none of risk assessment tools address all of the modifiable risk factors, nor do they consider non-modifiable risk variables. Patients who are receiving ICU level therapies that include artificial ventilation, Extracorporeal Membrane Oxygenation (ECMO), or vasopressors, and other particular demographics have additional risk factors that are not considered by the Braden Scale.

The Braden Scale is the most generally utilized scale [10,13,14,16,17,18,19,20,21]. Braden Scale comprises six separate subscales. Each subscale ranges from 1 (most risk) to 4 (least risk) with the exception of friction and shear which only ranges from 1 to 3. Consequently, the score range is between 6 and 23. A patient’s total score is the sum of his or her subscale scores. If the overall score on this scale is less than or equal to 18, the patient is anticipated to be at-risk for HAPI. If the overall score is more than 18, the patient is not considered at-risk [16].

Risk assessment tools are employed as a guide to assist nurses in identifying risk, but they are restricted by the number of factors that a caregiver may effectively consider during patient care. These evaluations also indicate a substantial proportion of hospitalized patients as being at risk for pressure injuries. Standardized risk assessments have the drawback that they necessitate additional interventions for a substantial proportion of the patient population who may not acquire pressure injuries.

The following are the disadvantages and restrictions of the risk assessment tools: There is no generic model that considers the aforementioned risk variables. Its False Positive Rate (FPR) is relatively high. Delivering interventions to the wrong patients will result in excessive intervention costs. Furthermore, risk assessment tools do not consider several related risk factors such as renal failure, Glasgow coma score, stimuli anesthesia, visiting ICU during hospitalization, weight loss, American Society of Anesthesiologists (ASA) Score, feeding tube, and many other risk factors as shown in Table 2.

Incorporating additional risk factors via Machine Learning (ML) approaches can help reduce the volume of patients identified as being at risk for pressure injuries. There is a need to identify the total level of risk using ML and risk assessment tools jointly and utilize this information to allocate resources toward individuals who are at the highest level of risk for pressure injuries.

### 1.6. Research Objectives

Researchers have paid considerable attention to PI through artificial intelligence in the last two decades because it is a quality-of-care measure that effects healthcare professionals’ responsibilities, the obligations of healthcare personnel, and patient care outcomes [22].

In the last 15 years, there is only one systematic review in the literature developed by Jiang et al. (2021) that describes how ML is utilized in PI management in 32 studies [23]. Their study discusses the three “main general” aspects of using ML without going into more depth in those studies. Therefore, a more in-depth analysis of the existing literature that centers around ML in PI management is lacking. Furthermore, there is no recent systematic review that summarizes and segments the studies of ML in PI management in the medical field.

This research primarily aims to address the contribution of existing literature in the past 15 years about the use of ML in PI management, categorize and discuss those studies into different sub-fields/trends of applications based on medical specialties, analyze existing gaps in the literature, and discuss opportunities for future applications and research directions. Specifically, this research focuses on “predicting HAPI before occurrence.”

### 1.7. Research Structure 

This research is organized as follows: Section 2 describes the methodology for collecting the reviewed papers, which includes the protocol, search strategy, inclusion and exclusion criteria, study selection methods, and data extraction. Section 3 reviews the results of study process, characteristics, and statistics of included studies, and categorizes reviewed studies into three groups and different trends within each group. Section 4 discusses, analyzes, and summarizes the three categories and trends, research gaps, and limitations. Section 5 discusses potential future applications and opportunities in PI management and addresses the limitations of the research method adopted in this review. Section 6 summarizes the conclusions of this review.

## 2. Methods

### 2.1. Reporting Method 

A systematic literature review is conducted by adopting Preferred Reporting Items for Systematic Reviews and Meta-Analyses (PRISMA) [24].

### 2.2. Search Strategy 

Four of the most popular databases were used in the systematic search: PubMed, Web of Science, Scopus, and Science Direct. Other individual searches were conducted by using the references of some eligible studies. The search was conducted in July 2022. All synonyms of pressure injuries were used in search terms. The asterisk was used to include any other likelihood for remaining potential related terms. For example, pressure injur* might include pressure injury, pressure injuries, pressure injuries risk assessment, pressure injuries prevention, pressure injuries management, or any other related terms. On the other hand, most of the ML and artificial intelligence terms are used. The search strategy and search terms are summarized in Box 1.

Box 1 **Search strategy.**

**(**Pressure injur* OR pressure ulcer* OR hospital-acquired pressure injur* OR bedsore* OR decubitus ulcer* OR bed sore* OR decubitus sore*)AND

**(**predictive modeling OR predictive analytics OR machine learning OR deep learning OR data mining OR early detection OR artificial* OR neural network OR convolutional neural network OR support vector OR random forest OR naïve OR logistic regression OR decision tree OR algorithm* OR regression OR k-nearest neighbor* OR multilayer perceptron OR adaboosting OR supervised learning OR unsupervised learning OR clustering OR K-means OR bayesian* OR gradient* OR natural language processing OR fuzzy logic OR computational* OR transfer learning**)**

### 2.3. Inclusion Criteria

The inclusion criteria are conducted to match the following conditions: (1) the published language is English; (2) the timeline is between January 2007 until July 2022; and (3) the study should include a method related to ML in pressure injury applications that include Neural Network (NN), Logistic Regression (LR), Multilayer Perceptron (MLP), k-Nearest Neighbor (KNN), AdaBoosting (AdaBoost), Decision Tree (DT), Naïve Bayes (NB), Support Vector Machine (SVM), Random Forest (RF), Extreme Gradient Boosting (XGBoost), Ridge Regression (RR), Logistic Regression (LR), Bayesian Additive Regression Tree (BART), Bayesian Network, Gradient Boosting, Linear Regression, Linear Discriminant Analysis (LDA), Light Gradient-Boosting Machine (LightGBM), any kind of Artificial Neural Network (ANN), any developed algorithm, any other algorithm(s), k-means, Self-Organizing Map (SOM) or any other clustering methods, Long Short-Term Memory (LSTM), Convolutional Neural Network (CNN), Region-Based Convolutional Neural Network (RCNN), Generative Adversarial Network (GAN), Recurrent Neural Network (RNN), any kind of Deep Neural Network (DNN), Principal Component Analysis (PCA), Least Absolute Selection and Shrinkage Operator (LASSO), Recursive Feature Elimination (RFE), and any dimensionality reduction algorithms, feature extraction/selection, Particle Swarm Optimization (PSO), Genetic Algorithm (GA), and any metaheuristic algorithms, Transfer Learning (TL), fuzzy logic, fuzzy sets and fuzzy theory, reinforcement learning, Natural Language Processing (NLP), Text Mining (TM), and Association Rule Mining (ARM).

### 2.4. Exclusion Criteria

Studies that match the following criteria were excluded: (1) any study that does not include ML or artificial intelligence; (2) dissertations, theses, case reports, editorials, conference abstracts, reports, opinion papers (i.e., any study that was not an article paper, review paper, or conference proceeding); (3) different kinds of ulcer (not pressure ulcer); (4) studies of pressure ulcers for animals; (5) studies that use only medical risk assessment tools, such as Braden Scale or Norton Scale; (6) studies that have unspecified wounds or general wounds; and (7) studies that have insufficient information or do not have clear outcomes.

### 2.5. Study Selection Methods

The above eligibility criteria were conducted by two independent reviewers by screening titles and abstracts. Then, full-text assessment and analysis were conducted. Any discrepancies were agreed upon by discussion.

### 2.6. Data Extraction

A full analysis is conducted for the included studies based on predefined criteria such as authors, year, country, objectives, methods, domain, publication type, source of publication, dataset size, inputs, and outputs. Then, based on the objective of the studies, studies are grouped into three segments. Extra analysis is conducted for the predictive analytics domain by extracting other information, such as the percentage of pressure injuries among datasets (i.e., it was calculated manually per each study), validation methods, performance metrics, algorithms, balancing methods, feature importance, feature selection, cost-sensitive learning, hyperparameters tuning methods, if the study is benchmark or retrospective study, and many others.

## 3. Results

### 3.1. Study Process

**Figure 4:**
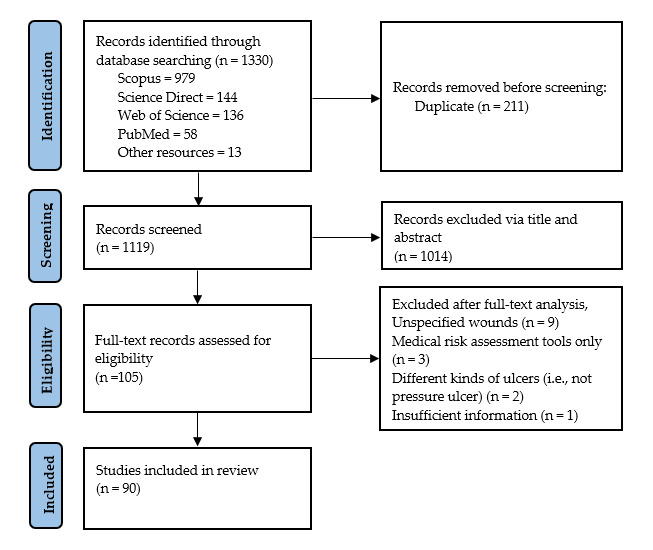
PRISMA flow diagram of the inclusion criteria.

The initial studies were 1330 published articles, with 211 duplications (979 studies from Scopus, 144 studies from Science Direct, 136 studies from Web of Science, 58 studies from PubMed, and 13 studies from individual searches conducted using the references of some eligible studies to ensure that most of the relevant studies were included). Then, 1014 studies were excluded after screening the titles and abstracts, which resulted in 105 potentially eligible studies for comprehensive analysis and assessment. Out of the 105 studies, 15 studies do not meet the eligibility criteria for the following reasons: (1) unspecified wounds (n = 9); (2) medical risk assessment tools only (n = 3); (3) different kinds of ulcer (i.e., arterial ulcers and venous ulcers, not pressure ulcers) (n = 2); and (4) insufficient information (n = 1). It results in 90 eligible studies for this review, as explained in Figure 4. 

### 3.2. Characteristics of Included Studies

**Figure 5:**
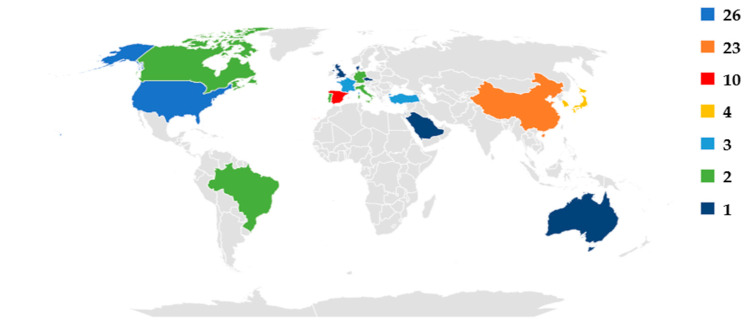
The studies included in this review per country.

**Figure 6:**
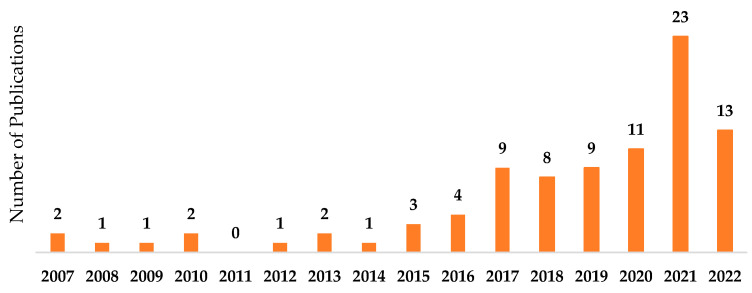
The studies included in this review per year.

There were a total number of 90 papers about ML in PI management published between January 2007 and July 2022. Figure 5 shows the number of published papers per country. Twenty-nine percent of the studies were published in the US (i.e., 26 studies), as shown in Figure 5; this is followed by China (26%), Spain (11%), Japan (4%), South Korea (3%), Turkey (3%), France (3%), Canada (2%), Brazil (2%), Germany (2%), Italy (2%), Portugal (2%), and other countries (11%). On the other hand, 76% of the publication were in journal papers, and 24% were published in conference proceedings. In the past 6 years, researchers’ interest in the investigated topics has steadily increased, as depicted in Figure 6.

### 3.3. Categorization of the Studies with High-Level Analysis

**Figure 7:**
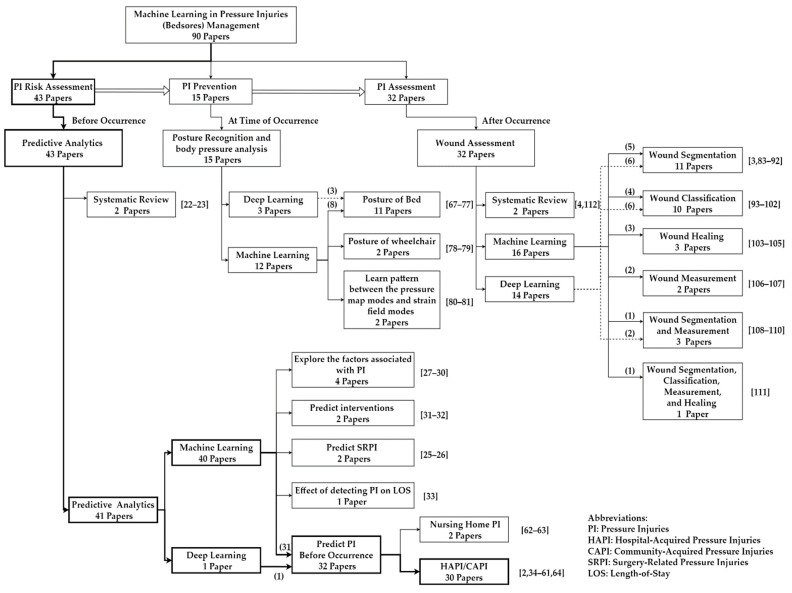
Flowchart of the three categories and sub-fields/trends of applications based on medical specialties of the reviewed studies.

The reviewed articles are divided into three categories based on the time of occurrence of PI: PI risk assessment, PI prevention, and PI assessment. To some extent, this structure is loosely based on Jiang et al. (2021) [23], which is then reviewed and further adjusted by the medical author. Different from Jiang et al. (2021), each major category is further broken down into sub-fields/trends of applications based on medical specialties, as summarized in Figure 7, which is one of the contributions of this review. 

**Figure 8:**
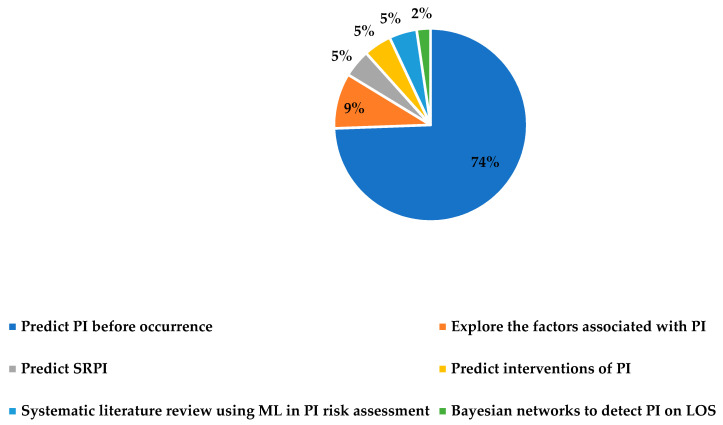
Distribution of investigated topics on PI risk assessment.

The first category is PI risk assessment (before occurrence), which is related to early intervention actions required by nursing before PI occurs, such as increased frequency of skin inspection for early identification of skin changes as well as assessment for non-visual cues, which include skin temperature, turgor, edema, or induration changes. Forty-three studies were conducted in this category. Seventy-four percent of the studies in the first category are concentrated on developing ML models to predict who will have PI/early prediction of HAPI, as summarized in Figure 8. Other predictive analytics models are related to: exploration of the factors associated with PI (9%), prediction of Surgery-Related Pressure Injuries (SRPI) (5%), prediction of types of interventions based on the conditions of the patients (5%), systematic literature review using ML techniques in PI risk assessment (5%), and outcome of adopting Bayesian networks to detect PI on Length-of-Stay (LOS) (2%).

**Figure 9:**
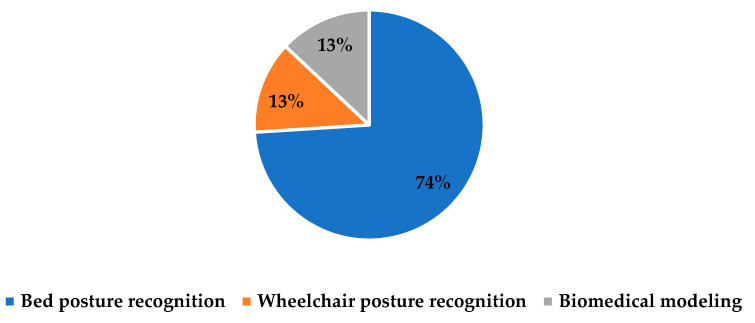
Distribution of investigated topics on PI prevention.

The second category is PI prevention (at time of occurrence), which is related to any intervention actions during PI by using ML to reduce it [23], such as turning patients every two hours, feeding the patient, using a lift to move in bed, and elevating heels [1]. Fifteen studies are classified under this category to predict posture recognition of the patients using ML and/or Deep Learning (DL) methods and therefore send a signal for nurses to provide a change to the patient’s position to reduce the likelihood of developing PI. Around seventy-four percent of the studies in the second category are concentrated on bed posture recognition; the rest of the studies are related to wheelchair posture recognition (13%) and biomedical modeling (13%), as summarized in Figure 9.

**Figure 10:**
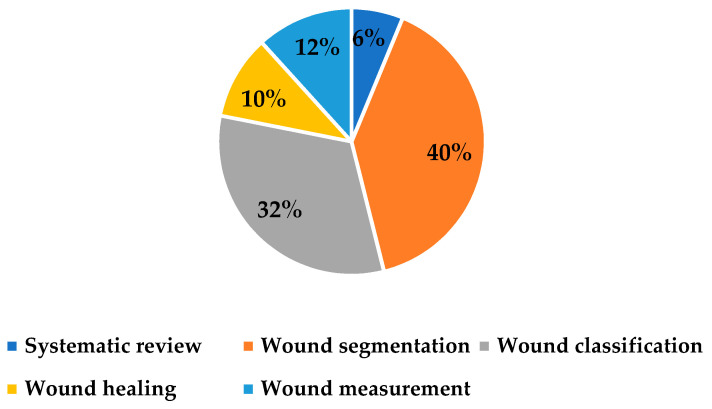
Distribution of investigated topics on PI assessment.

The third category is PI assessment (after occurrence), which is related to decisions to evaluate and measure the pressure injuries, such as staging of the wounds, monitoring wound healing, classification of wound types, and measuring the wounds, after PI occurs [4]. Thirty-two studies are classified under this category. Forty percent of the studies are related to wound segmentation; the rest of the studies are related to wound classification (32%), wound measurement (12%), wound healing (10%), and systematic review (6%), as summarized in Figure 10.

**Figure 11:**
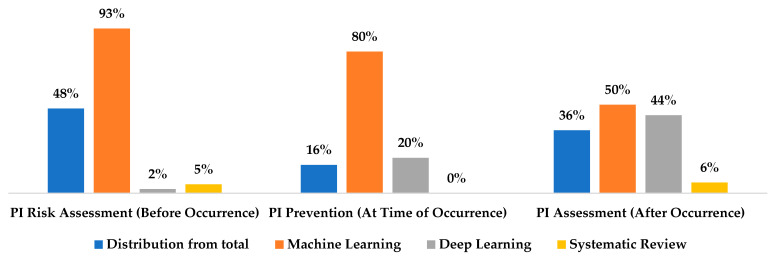
The three major categories of the studies included in this review.

Figure 11 shows that 48% of the studies were conducted on PI risk assessment (43 studies), 16% on PI prevention (15 studies), and 36% on PI assessment (32 studies). Figure 11 presents a high-level analysis for the studied review: ML was utilized in 93%, 80%, and 50% of the studies on PI risk assessment, PI prevention, and PI assessment, respectively. On the other hand, DL was used to analyze images in PI in 2%, 20%, and 45% of the studies on PI risk assessment, PI prevention, and PI assessment, respectively. Figure 11 illustrates an opportunity to use DL in all three categories (only one study was conducted on PI risk assessment, none conducted on PI prevention, and two studies were conducted on PI assessment). Furthermore, there is a need for a systematic review to explain the big picture of PI management. Nevertheless, a third of the 90 studies concentrated on predictive analytics of HAPI and CAPI. Therefore, this research focuses on predicting HAPI/CAPI before occurrence.

## 4. Discussion

This section discusses, analyzes, and summarizes the three categories of research about the use of ML in PI management. Each category is further broken down into sub-fields based on medical specialties. Research gaps and limitations are analyzed separately for each category. 

### 4.1. PI Risk Assessment (Before Occurrence)

Risk assessment refers to the early intervention actions that nurses need to take and complete before PI occurs. Use of a risk assessment tool, such as the Braden Scale, allows for identification of modifiable risk factors specific to the patient and allows the care provider to develop a plan of care that puts certain risk control interventions in place. The first Braden risk factor is sensory perception or the patient’s ability to respond meaningfully to pressure-related discomfort [1]. Interventions include increased frequency of skin inspection for early identification of skin changes as well as assessment for non-visual cues that include skin temperature, turgor, edema, or induration changes. The second Braden risk factor is moisture, the degree to which the skin is exposed to moisture; this factor is unique in its ability to affect both likelihood of increased mechanical load as well as affecting tissue tolerance [1]. Interventions focus on identifying and containing the source of moisture if moisture is unable to be contained; specialty surfaces and topical moisture barrier can be used to minimize exposure [1]. The third and fourth factors are activity and mobility limitations that assess the degree of physical activity and ability to change and control body position [1]. Interventions focus on increasing activity and limiting duration of pressure to bony prominences [1]. The fifth area assessed is the patient’s nutrition; nutrition affects the tissue tolerance [1]. Nutrition interventions focus on improving intake and minimizing disruptions to enteral feeding. The last area of focus for the Braden Scale is friction and shear; this area affects the likelihood of increased mechanical load [1]. Interventions for this area focus on reducing strain to the patient’s skin and tissue during periods of movement. Though the Braden Scale covers many of the modifiable risk factors, it does not address all of them nor does it consider non-modifiable risk factors. Risk assessments are used as a guide to help caregivers identify risk; however, they are limited by the number of factors that can reasonably be assessed by a caregiver at time of the assessment. Although these can be imperfect, they provide insight to which patients are at risk and guide resource allocation. 

Patients’ data in the Electronic Health Records (EHR) and some of the risk factors (Table 2) have been utilized in ML techniques to predict several issues related to PI risk assessment and to replace medical risk assessment tools such as Braden Scale. Table 3 summarizes the 43 papers published in this field that represent different applications/sub-fields, which include predicting SRPI [25,26], exploration of the factors associated with PI [27,28,29,30], prediction of types of interventions based on the conditions of the patients [31,32], and effect of adopting Bayesian networks to predict PI on LOS [33], systematic literature review using ML techniques in PI [22,23], and predicting PI before the occurrence [2,34,35,36,37,38,39,40,41,42,43,44,45,46,47,48,49,50,51,52,53,54,55,56,57,58,59,60,61,62,63,64].

Patients undergoing surgery are likely to develop PI during cardiovascular surgery. Predicting patients with SRPI is a challenging problem; extending this field of research by adopting state-of-the-art methods that may help increase prediction accuracy. Predicting patients with SRPI was conducted only twice in this section [25,26]. Those two studies utilized different algorithms on the same dataset, as shown in Table 3.

Exploring the factors associated with PI is an essential and elementary step to determine the significant risk factors that affect any PI (HAPI, CAPA, SRPI, or PI at a nursing home). Machine learning techniques, multivariate analysis, and univariate analysis can be applied to the historical records from EHR to discover the significant risk factors that might contribute to the development of PI. Those factors can be used as input to other prediction models to predict patients with PI [63]. This section has four studies [27,28,29,30]. Two studies explored factors that affect SRPI [27,29]. The other two studies explored the factors of PI for elderly patients and PI patients in general [28,30], respectively. It is worth mentioning that some studies explored the risk factors of PI by using risk factors in other studies in the literature [34,42]. Nonetheless, Table 1 and Table 2 in Section 1 summarize most risk factors that potentially contribute to the development of PI. 

Intervention actions according to the PI patients’ physical signs were predicted by utilizing different risk factors associated with PI, patients’ status, and physical characteristics [27,28,29,30]. This kind of research might be difficult to implement in practice because they predicted one action per patient. In some cases, patients require several actions that should be implemented concurrently, for instance, turning patients every two hours and improving intake and minimizing disruptions to enteral feeding. State-of-the-art methods can be utilized in this field by training a multi-task learning model that can provide different multiple actions. It is worth mentioning that PSO is used by Jin et al. (2021) in this domain as an optimization tool to optimize the hyperparameters of RF [31]. This was the only research among the 90 reviewed studies that used metaheuristics as an optimization tool for ML. There are opportunities for adopting metaheuristic algorithms with ML in all sub-field domains.

One research adopted Bayesian networks to evaluate the impact of the development of ML to predict patients with PI on LOS. This kind of prospective study is essential in the continuous improvement process to measure the effect of applying such a model in the hospital; the improvement is measured both before adoption of the predictive model and after. The same methodology can be utilized by measuring the effects of applying any predictive models in different Key Performance Indicators (KPIs), such as harm rate, PI rate, cost of prevention actions, and others.

There were two systematic reviews conducted by adopting PRISMA in this field [22,23]. Jiang et al. (2021) [23] analyzed 32 studies conducted in the field of ML in PI management. Their review included both Chinese and English languages in their review. Their study adopted several Chinese databases, such as the China Biomedical Literature Database (CBM), the Wanfang Database, and China National Knowledge Infrastructure (CNKI). The authors segmented the studies into three main topics: predictive analytics, posture recognition, and image analysis (image classification and measurement). The authors generally described the three topics without going into more depth in those studies. The authors misclassified many other essential topics, such as would segmentation of wound healing, biomedical models, predicting interventions, predicting SRPI, and exploring factors associated with PI, and other essential applications in PI. Their systematic review did not introduce potential new approaches and applications in this field. Therefore, there is a need for a more in-depth analysis of the existing literature and an updated systematic review about the use of ML in PI management, which is the objective of this research.

The second systematic review in this field was more specific; it was conducted by Ribeiro et al. (2021) [22]. The authors analyzed seven studies to explore the most relevant algorithms for PI prevention. The review provided a comprehensive analysis of results for each performance metric per study and compared them. Furthermore, the review provided the results for multiple algorithms per study. Their systematic review would be better if the authors included more studies and conducted analysis for several applications rather than PI prevention.

The last application in this category is predicting PI before the occurrence [2,34,35,36,37,38,39,40,41,42,43,44,45,46,47,48,49,50,51,52,53,54,55,56,57,58,59,60,61,62,63,64], which accounts for 36% (i.e., 32 studies) of the studies among the 90 reviewed studies. It relates to predicting patients who will develop PI before occurrence. There are two types of predictive models in this field. Each type of predictive models has different conditions and associated risk factors. The first type is predicting nursing home residents’ PI, which has two studies [62,63]. The second type is predicting HAPI/CAPI (i.e., which deals with patients in the hospital regardless of hospital-acquired pressure injuries or having PI on admission and then being admitted to the hospital with PI), which has 30 studies [2,34,35,36,37,38,39,40,41,42,43,44,45,46,47,48,49,50,51,52,53,54,55,56,57,58,59,60,61] and is the focus of this review.

Table 4 summarizes the research gap for all 30 published studies on predicting PI before its occurrence. Twenty-nine studies utilized ML to predict which patient will develop HAPI before it occurs by utilizing patients’ historical data in EHR [2,34,35,36,37,38,39,40,41,42,43,44,45,46,47,48,49,50,51,52,53,54,55,56,57,58,59,60,61]. In contrast, only one study, conducted by Wang et al. (2021), utilized DL on infrared thermal images of HAPI [64]. Wang et al. (2021) [64] trained and tested a CNN using 246 images; fifty percent were HAPI images and the other half were non-HAPI images. Their study was the first in this field using HAPI images. 

In terms of automation and implementation in hospitals, infrared thermal images of HAPI require several technical settings and procedures. Nonetheless, developing a prediction model using HAPI images is novel research. It can be advantageous to utilize 2D images of wounds to predict HAPI. Furthermore, Multimodal Machine Learning (MMML) can combine ML inputs (i.e., risk factors) and DL inputs (HAPI images) in one model to predict HAPI. The details of how to construct this hybrid model are explained in Section 5.4.1. 

Based on a thorough analysis of this field, there is no research that addresses when HAPI may occur in patients at risk. All studies in this field [2,34,35,36,37,38,39,40,41,42,43,44,45,46,47,48,49,50,51,52,53,54,55,56,57,58,59,60,61] answered the research question of which patient will develop HAPI, which provides the clinical team with insufficient information. Patients classified as at risk will likely continue to be at risk until discharged from the hospital. Therefore, a new study is needed to determine who will develop HAPI and when this development is likely to occur in patients at risk. 

Similarly, all the studies [2,34,35,36,37,38,39,40,41,42,43,44,45,46,47,48,49,50,51,52,53,54,55,56,57,58,59,60,61] adopted a single snapshot of patient status/conditions. Most of these records were collected on admission [34]. In this case, the predictive models do not capture the changes in patient’s status during hospitalization (i.e., from admission until HAPI). Therefore, there is a need for studies that utilize all patient status changes from admission to HAPI. The details of how to capture the changes in patient’s status are presented in Section 5.2.

Up till now, all the studies in this field [2,34,35,36,37,38,39,40,41,42,43,44,45,46,47,48,49,50,51,52,53,54,55,56,57,58,59,60,61] were mostly handled using classical ML or DL algorithms, as shown in Table 4 and Table 5. There were only two studies that used grid searches to tune the hyperparameters of ML models [34,45]. Metaheuristic algorithms were not addressed till now in this field to optimize the hyperparameters of ML models. Section 5.4.5 discusses how metaheuristic algorithms could be integrated with ML and/or DL to determine the best hyperparameters for ML models. 

As discussed in Section 1, HAPI is considered a “rare event.” In other words, the HA-PI rate of the patients who developed HAPI among all hospitalized patients is low (i.e., highly unbalanced dataset of HAPI and non-HAPI patients). Only five studies have a HAPI rate of less than or equal to 3% [34,39,46,59,60]. Those five studies adopted oversampling techniques to balance the highly unbalanced dataset, that includes Random Oversampling (RO), Under Sampling (US), and Synthetic Minority Oversampling Technique (SMOTE). Using those balancing methods, however, would likely overfit the model. Therefore, cost-sensitive learning is recommended to minimize the likelihood of overfitting in highly unbalanced datasets [65]. Nonetheless, there is only one study that adopted cost-sensitive learning [45] for an unbalanced dataset of HAPI (i.e., HAPI rate was 7.80%) [66].

Braden Scale was used as a standalone risk assessment tool in five studies that had a highly unbalanced HAPI rate [34,39,46,59,60]. Braden Scale covers many, but not all, of the modifiable risk factors; it does not consider non-modifiable risk factors. Critically ill patients, patients in the operating room, and other special populations have additional risk factors that are not addressed by the Braden Scale. Risk assessments are used as a guide to help caregivers identify risk; however, they are limited by the number of factors that can reasonably be assessed by a caregiver at time of the assessment. These assessments often identify a large percentage of hospitalized patients as being at risk. The disadvantage of using standardized risk assessments is the large percentage of at-risk patients that requires a lot of resources to mitigate the risk. Integrating ML with Braden Scale will allow for a larger set of modifiable risk factors (Table 2) to be considered in the prediction model, which can potentially achieve better model performance. The details of how to develop a hybrid ML-Braden Scale are presented in Section 5.1. 

Table 5 shows that the most commonly used algorithms in predicting PI were LR (19 times), RF (16 times), DT (12 Times), SVM (11 Times), MLP (10 Times), and KNN (4 times). The most common performance metrics were sensitivity, specificity, and Area Under the ROC Curve (AUC).

The limitation of the predictive models is that each model is designed based on patient records for a specific study, which means those models will require retraining if it is applied to a different population in another hospital. 

In summary, no more than 30 studies have been conducted in this field that answer who will develop HAPI among the patients without indicating when HAPI might occur. Twenty-nine studies adopted classical ML methods. One study adopted DL, whereas all the studies used a single snapshot of patient status without considering the effect of the changes in patients’ status during hospitalization. There are opportunities to implement state-of-the-art models in the literature to predict HAPI.

### 4.2. PI Prevention (At Time of Occurrence)

PI Prevention refers to actions that nurses need to take while patients are under PI. Those actions (i.e., care plan) include repositioning and turning the posture, feeding the patient, using a lift to move in bed, elevating heels, providing incontinence rounds, inspecting the skin frequently for signs of breakdown [1] or all other actions discussed in Section 3.1 to mitigate the likelihood of developing PI. Most of the studies use sensors to capture real-time data to track the changes of pressure in the body parts. Images of different postures are used to recognize the movement and positions of the at-risk patients.

Table 6 presents all ML and DL studies in PI prevention. This category includes posture bed recognition [67,68,69,70,71,72,73,74,75,76,77], posture wheelchair recognition [78,79], and a ML model is adopted to learn the pattern between pressure map modes and strain field modes [80,81].

This category is under development by researchers in labs and scientific centers [23]. Researchers have started to adopt ML concepts to images and signals. Most of the studies are prototypes [80,81] and used volunteers to gather the signals [67,68,69,70,71,72,73,74,75,76,77,78,79]. Such inputs must be trained and tested on patients instead of volunteers to get accurate results. The characteristics of patients with PI are statistically different from patients without PI [34,36,43,45,46,48,51,54]. Therefore, there is an opportunity to develop such studies using data from patients to initiate prevention actions for at-risk patients. It may be of interest to first predict patients at risk of developing PI (Section 4.1) and then determine preventive actions for the predicted at-risk patients with another model. Metaheuristics algorithms can be utilized to optimize the hyperparameters of ML and DL models during model development.

There is no systematic review that summarizes and discusses this field. Therefore, there is a potential for a systematic literature review with specific eligibility criteria to aggregate existing research in this topic and provides future opportunities for this field. 

In practice, the applications of this field can be complicated and may be infeasible to implement for many reasons. First, each bed requires a special kind of sensor (or device [81]) to detect the positions of the body, which can be costly and infeasible. Second, it requires human interactions and calibration to record the signals/inputs. Third, these inputs need to be continuously recorded in small time interval in real-time. 

### 4.3. PI Assessment (After Occurrence)

PI Assessment is related to any action after PI occurs. It focuses mainly on wound assessments that help understand the wounds’ characteristics through [4] (1) wound measurement, which measures the topology of the wounds such as surface area, wound size, and wound depth by analyzing 2D/3D images of the wounds; (2) wounds segmentation, which is related to selecting boundaries of the wounds among other tissues or it can be used in tissue identification; (3) wound classification, which is related to classifying wounds into different types (Figure 3), wound stages (Figure 2), or any related PI classifications; and (4) wound healing, which is related to the decision about the healing process of the wounds. Those characteristics will be used as input for a patient treatment plan.

When PI do develop prevention remains a critical strategy in treatment of the injuries, which focuses continuously on improving tissue tolerance and reducing the likelihood of increased mechanical load. PI treatment includes a holistic approach from identification through healing. The assessment of PI will include a comprehensive look at the patient to identify comorbidities as well as psychosocial factors that play a part in the wound’s ability to heal, this assessment will also establish treatment goals consistent with the patient’s wishes [1]. During the initial assessment baseline measurements and staging are obtained. PI staging identifies the greatest level of tissue in the wound bed and baseline measurements are needed as one factor to monitor wound healing [1]. With each subsequent visit the PI should be observed for wound changes that indicate a change in treatment is necessary; however, an injury should be allowed a two-week period to assess for progress towards healing [1]. Pressure Injuries are interesting because they begin as chronic wounds. Unlike pressure-related damage, an acute wound will follow the healing cascade and typically progresses through the four phases of healing in a month [82]. A pressure-related injury does not generally have a clearly identifiable mechanism of injury, such as surgery or traumatic event; the lack of moment of injury causes the wound healing cascade to begin in the inflammatory phase [82]. Chronic wounds do not follow an orderly and timely wound healing cascade, typically stall in the inflammatory phase as many of these wounds have large quantities of non-viable tissue that requires debridement [82]. These chronic wounds typically take months to years to heal; if the source of injury, in this case pressure, is not managed effectively these wounds may never heal. 

**Figure 12:**
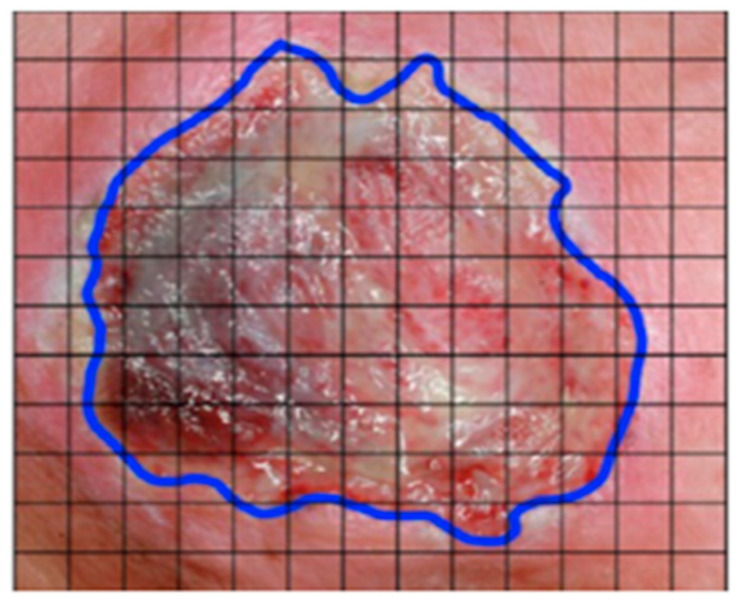
PI segmentation process ([109]).

Table 7 summarizes all ML and DL studies in PI assessment. This category’s four main medical areas are wound segmentation [3,83,84,85,86,87,88,89,90,91,92], wound classification [93,94,95,96,97,98,99,100,101,102], wound healing [103,104,105], and wound measurement [106,107]. Some studies had several targets/outputs within the same study, such as (1) wound segmentation and measurement [108,109,110] and (2) wound segmentation, classification, measurement, and healing [111]. An example of these hybrid models is determining the surface area of a PI wound (i.e., wound measurement) by calculating the area of small squares inside the blue boundaries (i.e., wound segmentation), as summarized in Figure 12 [109]. Another study developed a hybrid model of neural networks and Bayesian classifiers for wound segmentation [110]. 

Most of the studies utilized classical ML or DL techniques, as summarized in Table 7. However, only three studies adopted TL in wound assessments [87,93,96], whereas TL was not adopted in PI risk assessment (Section 4.1). TL can be utilized in this domain due to shortage of wound images, as PI is considered as a rare event [110].

It is worth mentioning that the pioneers of this research are Veredas et al. [90,92,105,110], Zahia et al. [3,4,108], and García-Zapirain et al. [88,89]. Their collective contribution to this field amounts to 28% of the published research. 

There is a potential to adopt DL in wound healing, but no studies in this sub-field has adopted DL methods. There are only three studies that used ML methods [103,104,105]. On the other hand, there is a potential for more wound measurement studies because only two studies in this field utilized ML approaches [106,107]. Nevertheless, there is an opportunity for a new updated wound assessment survey paper because it has been three years since the systematic review of Zahia et al. (2019) [4], whereas the second review conducted by Kaswan et al. (2020) [112] was a brief review of wound classification and segmentation, which analyzes 10 studies.

Only one study by Anisuzzaman et al. (2021) [96] adopted hybrid models that combine images of wounds and their corresponding location in MMML in wound classification: diabetic, pressure, venous ulcers, and surgical. There are no studies that adopted images of wounds and diagnoses of patients in one model (i.e., MMML) in wound segmentation, wound measurement, wound healing, and wound stages. Moreover, metaheuristics are not utilized in this field. Therefore, there are opportunities to improve classification accuracy by adopting a hybrid model between DL and metaheuristics. Besides, GANs are not applied in this field. Due to the scarcity of wound images (i.e., PI is considered as a rare event), GANs can be utilized to generate new wound instances rather than using traditional augmentation techniques [113,114].

Predicting stages of PI is not equivalent to the prediction of when PI happens. For instance, the first stage of PI might happen after a month of being admitted or after a week. Similarly, stage 4 might happen within a few weeks of hospitalization or it might take a few months. Therefore, predicting the stages of PI does not help nurses differentiate the urgency of those predicted stages. Therefore, there is a need to have a way to predict when PI happens for those who would develop PI, then provide an early preventive action for those who will be more likely develop PI within a specific timeframe (i.e., highest risk patients).

Lastly, there is no research focus on the holistic approach of the patient’s journey from admission to discharge that includes PI risk assessment (before occurrence), PI prevention (at time of occurrence), and PI assessment (after occurrence). Each research is conducted in an isolated area. There are opportunities for research that integrates two or more areas—for example, prediction of PI before the occurrence and prediction of the intervention actions when PI happens for at-risk patients based on the characteristics of wounds and risk factors.

## 5. Potential Future Opportunities

The previous contributions in PI were mostly addressed using classical ML techniques and applications. Based on the in-depth analysis of the previous studies, there are opportunities for new applications in PI management that will help the clinical team better utilize available resources and apply state-of-the-art models in another field in PI management. This section describes the most relevant and potentially useful applications and methods in PI management, and discusses the limitations of the research method adopted in this review.

### 5.1. Integrating Braden Scale with Machine Learning 

As discussed in Section 4.1, although the Braden Scale covers many of the modifiable risk factors, it does not address all of them nor does it consider non-modifiable risk factors. The integration of ML that considers all risk factors (Table 2) with Braden Scale with its sub-scales (sensory perception, moisture, activity, mobility, nutrition, and friction/shear) will potentially lead to better risk assessment of PI. The economic impact of such model can be quantified in terms of the cost reduction in preventive actions and resources needed to provide to patients identified as at-risk (savings in terms of FPR, i.e., wrong target). The National Pressure Injury Advisory Panel (NPIAP) reports that the average cost of prevention is $ 50–100 daily per patient in terms of time, pressure reducing support surfaces, labor, devices, and products [115].

**Figure 13:**
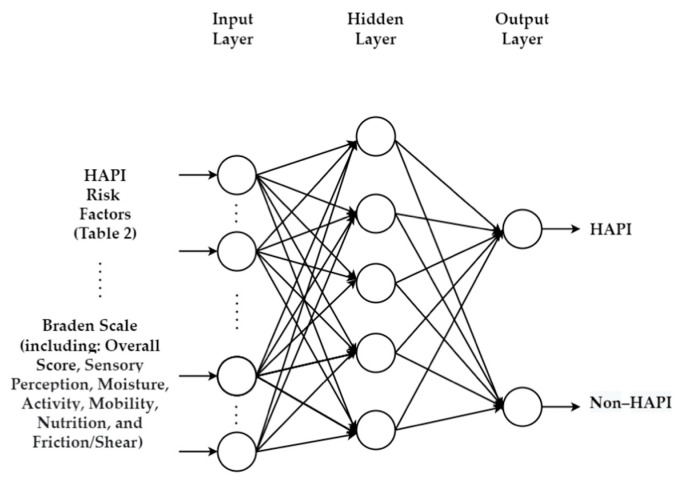
Integrated structure of MLP with Braden Scale.

An example of an MLP with Braden Scale to predict HAPI is illustrated in Figure 13. Other ML approaches can be used to replace MLP in such illustration. This type of model can be adopted for several applications, such as predicting intervention actions, predicting SRPI, predicting PI before occurrence for home residents/HAPI/CAPI, predicting healing evaluation, and others. 

### 5.2. Utilizing Real-Time/Daily Electronic Health Records 

**Figure 14:**
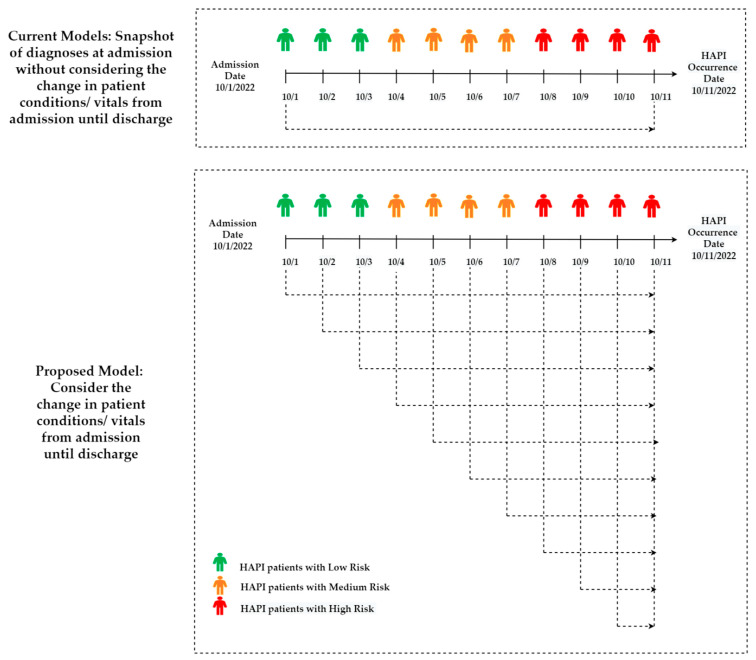
Proposed model to predict PI using real-time diagnoses.

Until now, all of the predictive models discussed in Section 4.1 and Section 4.3 used a single snapshot of patient records/risk factors/status/conditions. Such models do not capture the changes in the patient’s status during hospitalization (i.e., from admission until HAPI). Figure 14 illustrates how to utilize all patient status changes from admission to HAPI. The illustration provides an example of the difference in structure between the current models that have one diagnosis at admission for a patient at low risk versus a well-structured model that captures 11 different diagnoses for the same patient by changing the risk status during hospitalization. The proposed model more realistically simulates the current situation of admitted patients, as their status often change from admission to HAPI. The proposed model can be utilized for several applications of PI predictive analytics, such as predicting interventions, predicting SRPI, and predicting PI before occurrence for home residents/HAPI/CAPI.

**Figure 15:**
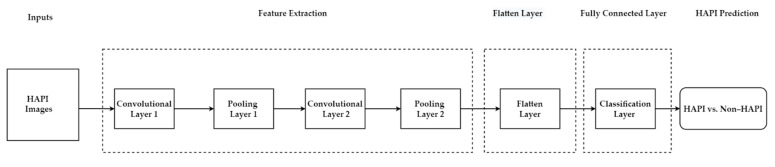
CNN to predict HAPI using real-time wound images.

The proposed model in Figure 14 can utilize only the risk factors (Table 2), the integrated ML-Braden Scale model (Section 5.1), or real-time images of wounds to predict HAPI or any other related targets/outputs. Figure 15 provides an example of how real-time images of HAPI can be used to develop a CNN to predict HAPI. Alternatively, other ML approaches can be used for the feature extraction task, followed by another ML model to predict HAPI.

### 5.3. Predicting Multiple Targets/Outputs

**Figure 16:**
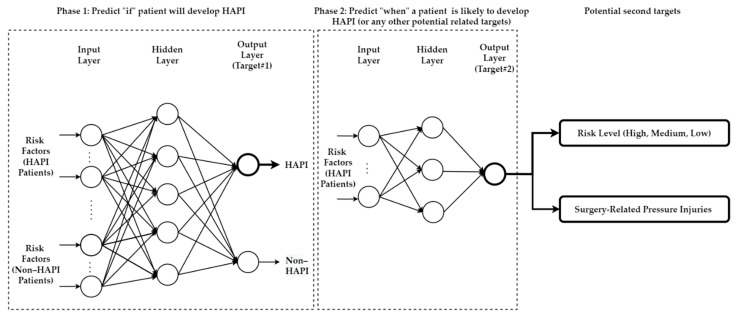
Proposed model structure of MLPs to predict multiple targets.

Multiple targets can be predicted simultaneously—all the current models used single-task learning by predicting one target, as explained in Section 4. However, a second target can be added, e.g., risk level or SRPI, as illustrated in Figure 16**.** MLPs are included in Figure 16 as a sample model structure for predicting multiple targets. Other ML approaches can be used to replace MLP in the illustration. 

All current models predicted only HAPI. However, predicting who will develop HAPI in the future cannot satisfy the degree of risk of those at-risk patients. All of them will be treated equally likely in terms of the risk level. Therefore, identifying the risk levels of at-risk patients can help stratify patients’ risks and provide prevention actions for those at the highest risk. As another example, models can predict who will develop HAPI and which of them will develop HAPI during surgery.

### 5.4. Hybrid Models

**Figure 17:**
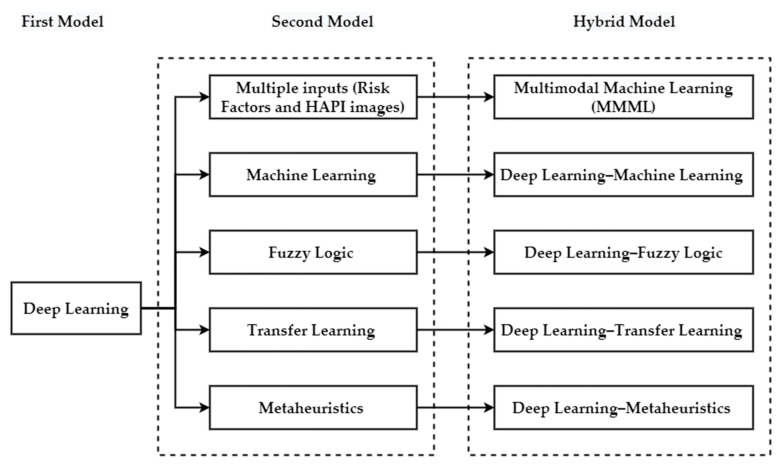
Potential integrated system between DL and other artificial intelligence methods to manage PI.

Deep learning can be integrated with one or more existing artificial intelligence methods to potentially enhance the method’s performance, reduce the noise in the dataset, reduce the complexity and computational time, and avoid overfitting [116]. As explained in Section 4, there is a gap in integrating models to predict HAPI or other related targets. Therefore, hybrid DL can be redesigned to take in several types of inputs, such as HAPI wounds images and/or metadata/risk factors in the case of PI. Fuzzy logic, metaheuristics, TL, ML, and MMML can be integrated with DL to manage PI, as shown in Figure 17. There are other possibilities for complex hybrid model that include (1) integrating two DL models together, such as RNN with CNN [117], Visual Geometry Group (VGG) based NN, and Spatial Transformer Network (STN) with CNN [118]; (2) hybrid system of DL, ML, and TL [119,120,121,122]; (3) hybrid systems of feature extraction using CNN, GA, and MLP [123]; and (4) hybrid system of ML and ARM [124]. The following sections briefly discuss how DL can be integrated with other models to manage PI. 

#### 5.4.1. Multimodal Machine Learning

**Figure 18:**
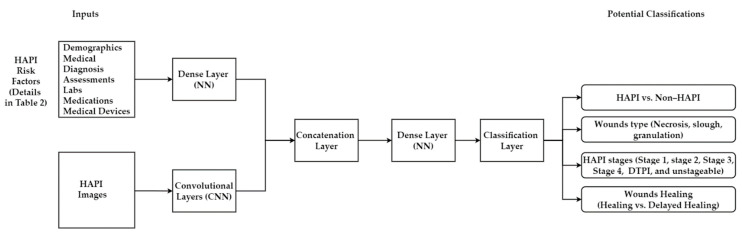
Combining PI images with risk factors to classify PI.

This hybridization combines ML inputs (i.e., risk factors) and DL inputs (HAPI images) in one model. First, the PI images will be fed to a CNN for processing (feature extraction from the images). At the same time, HAPI risk factors will be fed to a NN to extract the relevant features from the risk factors. Then, the extracted features from PI images and HAPI risk factors will be combined in one layer, as shown in Figure 18. After that, it will be treated as one problem (one layer with all extracted features). The Concatenation Layer will go to a Dense Layer (i.e., fully connected) that helps learn the hidden relationship in the extracted features. Lastly, the Classification Layer will provide potential classifications for PI. MMML has been widely used to classify skin lesions [125,126,127,128], COVID-19 [129], Alzheimer [130], and burn surgical [131] in the last few years.

#### 5.4.2. Deep Learning-Machine Learning Hybrid Model

**Figure 19:**
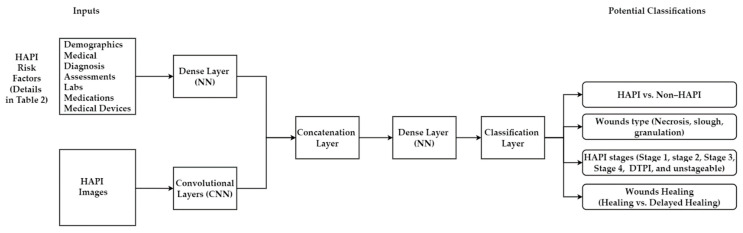
Hybrid deep learning—machine learning to classify PI.

This hybridization combines DL and ML into one structure. First, HAPI images will be fed into a CNN to extract the features (i.e., feature extraction and dimension reduction). Then, the extracted features will be fed to a ML layer (classification layer) by utilizing the power of the ML classifiers, such as SVM, to classify different potential targets of PI, as shown in Figure 19. This kind of hybrid model offers promising results in diagnosing lung cancer [132], detecting glaucoma [133], and detecting brain tumor [134]. 

#### 5.4.3. Deep Learning-Fuzzy Logic Hybrid Model

**Figure 20:**
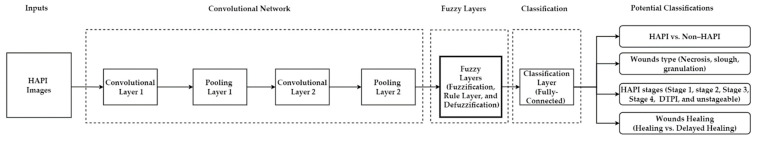
Hybrid deep learning—fuzzy logic to classify PI.

Fuzzy logic can add extra flexibility to the DL structure and enhance the model’s prediction accuracy [135,136,137,138]. After the features are extracted from HAPI images by a CNN, the extracted features will be fed to Fuzzy Layers. The input (i.e., extracted features) is converted from crisp numbers to fuzzy values using membership functions [139]. Then, the underlying rules are learned through the Rule Layer [139]. Thereafter, the Fuzzy Output Layer (i.e., Defuzzification) converts the fuzzy values back to a crisp form [135,139]. The output from the Fuzzy Layers will then be fed to a Fully Connected Layer for classifications, as illustrated in Figure 20. This concept can be utilized in PI management to enhance the accuracy of PI classification. Hybrid models of DL and fuzzy logic have been widely utilized in the medical field in the last few years, such as detecting tumors [140], detecting COVID-19 [141,142], melanoma diagnosis/skin lesion [143,144,145], and detecting breast cancer [146,147].

#### 5.4.4. Deep Learning-Transfer Learning Hybrid Model

**Figure 21:**
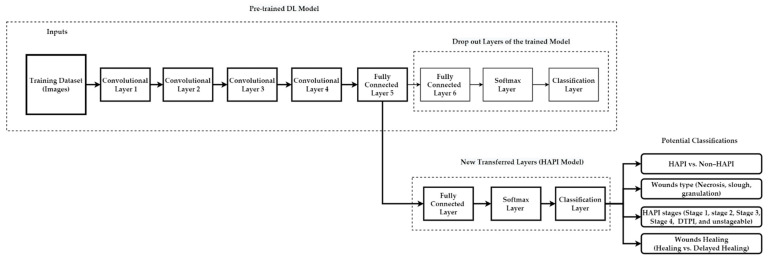
Hybrid TL-CNN to classify PI.

Transfer learning can take advantage of the information learned (trained weights/best features) in one well-trained model and transfer the knowledge to another model [116,148]. TL can be used to predict HAPI by using a pre-trained DL initial model to train a HAPI model. As an example, the methodology drops out the last three layers of the pre-trained model (i.e., Fully Connected Layer 6, Softmax Layer, and Classification Layer) and replaces them with three new layers of the HAPI model (i.e., use the parameters/weights from a well-trained model as a starting point to train a CNN) to predict potential classification targets of PI, as illustrated in Figure 21. Because HAPI is considered a “rare event” in hospitals (i.e., a small number of available HAPI images), TL can be utilized in this scenario due to the scarcity of available images. A hybrid system of TL with DL has been widely used in prediction in the medical field and provides reasonable performance, such as detecting skin cancer [149], detecting pathological brain [150], and detecting breast cancer [151]. Zero-Shot Learning (ZSL) can also be utilized in this field [152].

#### 5.4.5. Deep Learning-Metaheuristics Hybrid Model

In DL, many algorithms, such as CNNs, are used. Each has its parameters/settings/input to develop the algorithm. For example, CNN has many parameters, such as the number of kernels and size of the kernels in the convolutional layers and pooling layers, stride, padding, activation functions, and termination criteria [153]. The rule of metaheuristics is to work as an optimizer (simulator) in conjunction with CNN to find the best hyperparameters that provide a decent performance metric. One of the most popular metaheuristics used with DL is GA. GA is a robust stochastic population search technique that examines large search areas efficiently (global search). Similar to GA, other metaheuristics can be used in this kind of research.

Metaheuristics can be integrated with CNN or any classification method to classify potential targets of PI. Examples of Metaheuristic algorithms are GA, Ant Colony Optimization (ACO), Simulated Annealing (SA), Tabu Search (TS), Particle Swarm Optimization (PSO), Cuckoo Search (CS), and many others. Integrating metaheuristics with DL or ML has been proven to have a decent classification accuracy in the last decades; examples are cervical cancer diagnosis [154,155], COVID-19 [123,156,157,158], and anemia disease [159].

### 5.5. Limitations of Research Method Adopted in This Review

This research has some limitations. It considered only publications in conference proceedings and journals by excluding other types of publications such as dissertations, case reports, and conference abstracts, which include relevant studies in dissertations and other materials could be better. Any publication written in any language except English was excluded. This research used four databases, which can be extended in the future to include other databases, such as Ovid MEDLINE, EMBASE, Europe BMC, CINAHL, Wiley Online Library, and IEEE Xplore. On the other hand, each study adopted one or several algorithms, each with different performance metrics. Therefore, this review focused on highlighting the settings, inputs, and characteristics of the methodologies rather than the results of algorithms because study results depend on the characteristics of patients, and none of the designed models can be generalized for all patients in all settings. The reviewed articles were divided into three categories based on the time of occurrence of PI. However, the division of studies according to the area of application could be added, such as ICU, neurosurgery, spinal cord injury, and ECMO. Lastly, the inclusion and exclusion criteria were designed to be general for all studies, after which three categories and 16 research trends were discovered after analyzing the studies. However, it will be better to design specific inclusion and exclusion criteria per each discovered trend to provide a more accurate segmentation of the discovered trends.

## 6. Conclusions

Machine learning has endless applications in the field of healthcare. When the EHR first became available, many healthcare practitioners fought against its adoption, fearing the high cost of digitizing medical records would outweigh any benefits and would lead to a depersonalized approach to healthcare. Today, fears still persist, in which nurses and doctors cite spending more time at the computer than with the patient. However, the strides made in digitizing patient health records has led to data that can be easily retrieved and reviewed to improve the delivery of healthcare and provide better overall patient care. 

Application of ML techniques to available data can ‘see’ trends in the data that a manual review could never hope to achieve. Specifically related to prevention of PI, ML can partner with or replace current manual risk assessments performed by the bedside caregiver. Current manual risk assessments performed by the care team at the bedside are limited to the amount of information that is easily assessed during bedside care; currently most are fewer than 10 assessment fields in a combination of objective and subjective data points. Machine learning can assess greater data points as well as monitor changes in the assessment over time, which looks at the patient in a holistic way that would not be possible in a manual risk assessment performed at prescribed intervals. 

The potential exists for further DL techniques and hybrid models. Integrating the assessment scales with ML can lead to a partnership between care providers and technology. Using the subjective data that can only be obtained from patient interaction with the data available to identify trends and changes in care needs, an unintended benefit of this type of partnership is the reduction in discrepancies from shift to shift or data getting ‘lost’ during hand off. The development of an EHR system was initially intended to be a tool to allow data transfer between providers and have patient data readily available for providers. The future of healthcare is a partnership between providers and technology to identify gaps, trends, and support the individualized care of the patient. 

## Figures and Tables

**Table 1:** The most commonly used risk assessment tools for predicting PI (adapted from [1]).

The Most Common Risk Assessment Tools		Braden Scale	Waterlow Scale	Norton Scale	Cubbin-Jackson Scale	Braden Q Scale	SCIPUS
**Specialty**		**General**	**General/Orthopedics**	**General/Elderly** **Patients**	**ICU Patients**	**Pediatric** **Patients**	**Spinal Cord**
**Strengths**	**High Sensitivity**	**✕**	**✕**	**✕**	**✕**	**✕**	**✕**
	**Generalizability**	**✕**		**✕**			
**Drawbacks**	**Limited Number of Risk Factors**	**✕**	**✕**	**✕**	**✕**	**✕**	**✕**
	**High False Positive Rate**	**✕**	**✕**	**✕**	**✕**	**✕**	**✕**
**Risk Factors**	**Skin Status**		**✕**		**✕**		
	**Mobility**	**✕**	**✕**	**✕**	**✕**	**✕**	**✕**
	**Friction Shear**	**✕**					**✕**
	**Blood Glucose Levels**						**✕**
	**Hygiene**				**✕**		
	**Activity Status**	**✕**		**✕**		**✕**	
	**Hemodynamics**				**✕**		
	**Perfusion**		**✕**			**✕**	
	**Cardiac Disease**						**✕**
	**Oxygenation**				**✕**	**✕**	
	**Tobacco Use**						**✕**
	**Gender**		**✕**				
	**Neurological Deficit**		**✕**				
	**Poor Nutrition Status**	**✕**	**✕**	**✕ ✓**	**✕**	**✕**	
	**Age**		**✕**		**✕**		**✕**
	**Sensory Perception**	**✕**				**✕**	
	**Incontinence/Continence**		**✕**	**✕**	**✕**		**✕**
	**Respiration**				**✕**		
	**Increase Skin Moisture**	**✕**				**✕**	
	**Abnormal Lab Blood Results**						**✕** #
	**Medications**		**✕**				
	**Renal Disease**						**✕**
	**Mental Condition**			**✕**			**✕**
	**Respiratory Disease**						**✕**
	**Weight for Height**		**✕**				
	**Physical Condition**			**✕**			
	**Past Medical Condition**				**✕**		
	**Major Surgery/Trauma**		**✕**		**✕**		

#: Hematocrit and Albumin; ✓: modified scale.

**Table 2:** Significant risk factors affecting PI by all other traditional (medical) risk assessment tools.

Demographics	Medical	Diagnosis	Assessments	Labs	Medications	Medical Devices
Age	Admission Source	Comorbidity	Blood Pressure Systolic	Albumin	Opioids	Artificial Air Management
Ethnic Group	American Society of Anesthesiologists (ASA) Score	Depression	Blood Pressure Diastolic	Blood Urea Nitrogen (BUN)	Steroid Use	Face Mask
Race	Length-of-Stay at Emergency Department	Diabetes	Body Mass Index (BMI)	C-reactive Protein	Stimuli Anesthesia	Nasal Cannula
Sex	Visiting ICU during Hospitalization	Pressure Injury on Admission	Count of Glasgow Coma Score (GCS) Comment	Creatine Serum	Stimuli Paralytics	Noninvasive Ventilation
	Number of Surgeries	Renal Failure	Glasgow Coma Score	Hemoglobin	Stimuli Sedation	Pharyngeal
	Number of Pressure Injuries	Sepsis Diagnosis	Weight Loss	High Mean Arterial Pressure (MAP)	Stimuli Tracheostomy	Room Air
	Palliative Orders	Stroke History	Patient Refusal to Change Position	Lactate	Vasopressor	Ventilator
	Prior Year Inpatient Visit Counter		Pulse Oximetry	Sodium		Feeding Tube
	Steroid History		Skin Abnormality on Admission			
	Visiting Transitional Unit during Hospitalization		Body Temperature			

**Table 3:** Sub-fields of applications based on medical specialties in PI risk assessment.

* Area	Reference		Author		Year	Country	Type	Dataset Size	Algorithm/ Method	Validation		Input of the Model		Output of the Model
**Prediction of SRPI**	[25]		Cai et al.		2020	China	Journal	149 patients	XGBoost	N/A		Risk factors for those who underwent cardiovascular surgery		Predict patients who will develop SRPI
	[26]		Chen et al.		2018	China	Journal	149 patients	ANN	N/A		Risk factors for those who underwent cardiovascular surgery		Predict patients who will develop SRPI
**Exploration of the factors associated with PI**	[27]		Aloweni et al.		2019	US	Journal	269 patients	Multivariate LR	N/A		Potential risk factors for those who underwent operations		Identify risk factors associated with patients who will develop SRPI
	[28]		Moon and Lee		2017	South Korea	Journal	15,856 patients	DT, univariate analysis	10-fold CV		Records from the Health Insurance Review and Assessment (HIRA Service), and National Inpatient Sample (NIS)		Identify risk factors associated with elderly patients who will develop PI
	[29]		Lu et al.		2017	China	Journal	149 patients	LR, univariate analysis, and multivariate analysis	N/A		Potential risk factors for those who underwent operations		Identify significant risk factors associated with patients who will develop SRPI
	[30]		Raju et al.		2015	US	Journal	1653 patients	LR, DT, RF, Multivariate adaptive regression splines	10-fold CV		Potential risk factors for PI patients that include Braden Scale, lab values, and demographics		Identify risk factors associated with patients who will develop PI
**Prediction of types of** **interventions based on the conditions of the patients**	[31]		Jin et al.		2021	China	Conference	1483 patients	PSO-RF, KNN, SVM DT	10-fold CV		Different risk factors associated with PI patients/ patient status and physical characteristics		Predict the treatment/action needs for patients with PI based on risk factors (i.e., guidelines for actions)
	[32]		Mota et al.		2019	Portugal	Conference	1339 patients	DT, NB	66.00% Training vs. 34.00% testing		Different risk factors associated with PI patients/ patient status		Predict the treatment/action needs for patients with PI based on risk factors (i.e., guidelines for actions)
**Outcome of adopting Bayesian networks to** **detect PI on LOS**	[33]		Cho et al.		2013	South Korea	Journal	1214 Patients	Bayesian Networks	N/A		EHR of patients with PI		Evaluate the impact of the development of ML model to predict patients with PI (i.e., improvement before adopting a predictive model and after)
**Systematic literature PI** **management**	[23]		Jiang et al.		2021	China	Journal	32 Studies	A systematic review was conducted by analyzing 32 studies related to ML in PI management (PRISMA)					
**Predicting PI before** **occurrence**	Systematic literature	[22]	Ribeiro et al.		2021	Portugal	Journal	7 Studies	A systematic review was conducted by analyzing seven studies (PRISMA) to discover the most related algorithms for PI prevention					
	Predicting PI for nursing home residents	[62]	Charon et al.		2022	France	Conference	3000 Patients	Bayesian networks, RF		N/A		Nursing home patients’ variables	Predict who will develop PI in nursing home
		[63]	Lee et al.		2021	South Korea	Journal	60 Patients	RF, LR, SVM		N/A		Nursing home patients’ variables	Predict factors for PI related to nursing home residents and predict PI
	Predicting HAPI/CAPI		ML	[2,34,35,36,37,38,39,40,41,42,43,44,45,46,47,48,49,50,51,52,53,54,55,56,57,58,59,60,61]	Table 4 and Table 5 summarize the 30 studies that had the same inputs (risk factors) and same outputs (i.e., predicting PI before occurrence (HAPI/CAPI))									
			DL	[64]										

* All studies adopted ML except [22,23,64]; CV: Cross-validation; N/A: Not mentioned; Conference: Conference paper.

**Table 4:** Studies that used ML and DL to predict HAPI/CAPI before occurrence from January 2007 till July 2022.

Study	Author(s)	Year	Country	Type	Dataset Size	Validation Method	Feature Importance	Feature Selection	Cost-Sensitive Learning	Hyperparameter Tuning (Grid Search)	Balancing Method	HAPI %	Retrospective Study	Comparing Results to Risk Assessment Tools
[53]	Šín et al.	2022	Czech Republic	Journal	9304	80% Training vs. 20% testing	**✓**				RO	50.00		
[54]	J. Xu et al.	2022	China	Journal	618	70% Training (5-fold CV) vs. 30% testing					RO	33.33	**✓**	**✓**
[55]	Do et al.	2022	US	Journal	6742	70% Training (5-fold CV) vs. 30% testing		**✓**			RO	31.92	**✓**	**✓**
[35]	Walther et al.	2021	Germany	Journal	149,006	10-fold CV	**✓**	**✓**			RO	3.10	**✓**	
[34]	Nakagami et al.	2021	Japan	Journal	75,353	70% Training (5-fold CV) vs. 30% testing	**✓**	**✓**		**✓**	RO	0.52	**✓**	
[2]	W. Song et al.	2021	US	Journal	188,512	80% Training (5-fold CV) vs. 20% testing	**✓**	**✓**			RO	3.27	**✓**	
[36]	Alderden et al.	2021	US	Journal	5101	80% Training vs. 20% testing					SMOTE	6.50	**✓**	**✓**
[37]	Anderson et al.	2021	US	Journal	23,000	80% Training vs. 20% testing					SMOTE	N/A	**✓**	**✓**
[47]	Ahmad et al.	2021	US	Conference	713	10-fold CV		**✓**			RO	52.31		
[38]	Ossai et al.	2021	Australia	Conference	1014	10-fold CV		**✓**			SMOTE	N/A	**✓**	
[48]	J. Song et al.	2021	China	Journal	5814	50% Training (10-fold CV) vs. 50% testing		**✓**			US and RO	28.78	**✓**	
[64]	Y. Wang et al.	2021	China	Journal	246	67% Training vs. 33% testing		**✓**			Image rotation and image dilation	50.00	**✓**	
[61]	Cheng et al.	2021	China	Journal	245	5-fold CV	**✓**				US	80.00	**✓**	
[39]	Hu et al.	2020	China	Journal	11,838	50% Training (10-fold CV) vs. 50% testing	**✓**	**✓**			US and RO	1.36	**✓**	
[40]	Vyas et al.	2020	US	Conference	13,282	80% Training vs. 20% testing					RO	16.80		**✓**
[44]	Ladios-Martin et al.	2020	Spain	Journal	6694	64% Training vs. 36% testing					SMOTE	4.12	**✓**	**✓**
[59]	Levy et al.	2020	US	Journal	57,227	80% Training (5-fold CV) vs. 20% testing	**✓**	**✓**			SMOTE, RO	0.42	**✓**	
[45]	Cramer et al.	2019	US	Journal	50,851	80% Training vs. 20% testing		**✓**	**✓**	**✓**	SMOTE, RO	7.80		**✓**
[57]	Hyun et al.	2019	US	Journal	12,654	N/A					RO	5.81	**✓**	**✓**
[51]	H. L. Li et al.	2019	China	Journal	2062	K-fold CV (k is not specified)					RO	50.00	**✓**	
[52]	Cichosz et al.	2019	Denmark	Journal	383	65% Training (5-fold CV) vs. 35% testing		**✓**			RO	28.10	**✓**	**✓**
[42]	Alderden et al.	2018	US	Journal	6376	67% Training vs. 33% testing					RO	8.10	**✓**	
[46]	Gao et al.	2018	China	Journal	1963	N/A					RO	2.38	**✓**	
[56]	Kaewprag et al.	2017	US	Journal	7717	67% Training vs. 33% testing	**✓**	**✓**			RO	7.65	**✓**	**✓**
[41]	Y. Jin et al.	2017	South Korea	Journal	11,191	80% Training vs. 20% testing		**✓**			RO	20.00	**✓**	**✓**
[43]	Deng et al.	2017	China	Journal	468	10-fold CV		**✓**			RO	20.10	**✓**	**✓**
[60]	Setoguchi et al.	2016	Japan	Journal	8286	10-fold CV	**✓**		**✓**		N/A	0.62		**✓**
[50]	Su et al.	2012	China	Journal	168	4-fold CV		**✓**			RO	4.80	**✓**	
[49]	Y.-C. Chen et al.	2008	China	Conference	168	3-fold CV					RO	4.80	**✓**	
[58]	Borlawsky and Hripcsak	2007	US	Journal	3151	4-fold CV		**✓**			RO	8.10	**✓**	

RO: Random Oversampling; US: Under Sampling; SMOTE: Synthetic Minority Oversampling Technique; CV: Cross-Validation; N/A: Not Mentioned; Conference: Conference Paper.

**Table 5:** Performance metrics and algorithms used to predict HAPI/CAPI before occurrence from January 2007 till July 2022.

Study	Performance Metrics								Algorithm Adopted							
	Accuracy	Sensitivity	Specificity	Precision	FI-score	AUC	False Positive	Other(s)	LR	RF	DT	SVM	MLP	KNN	LDA	Other(s)
[53]	**✓**	**✓**		**✓**	**✓**	**✓**	**✓**	**✓**	**✓**	**✓**		**✓**	**✓**	**✓**		**✓**
[54]	**✓**	**✓**	**✓**			**✓**		**✓**	**✓**	**✓**	**✓**					
[55]		**✓**	**✓**			**✓**			**✓**	**✓**	**✓**			**✓**		**✓**
[35]						**✓**			**✓**							**✓**
[34]		**✓**	**✓**			**✓**	**✓**	**✓**	**✓**	**✓**		**✓**				**✓**
[2]	**✓**	**✓**	**✓**		**✓**	**✓**			**✓**	**✓**		**✓**	**✓**			
[36]						**✓**				**✓**			**✓**			**✓**
[37]	**✓**	**✓**	**✓**		**✓**		**✓**	**✓**	**✓**	**✓**						
[47]	**✓**	**✓**		**✓**	**✓**	**✓**			**✓**							**✓**
[38]		**✓**	**✓**	**✓**			**✓**	**✓**		**✓**	**✓**		**✓**	**✓**	**✓**	
[48]	**✓**	**✓**		**✓**	**✓**	**✓**				**✓**	**✓**	**✓**	**✓**			
[64]	**✓**	**✓**	**✓**			**✓**				**✓**		**✓**				**✓**
[61]	**✓**									**✓**		**✓**	**✓**			**✓**
[39]		**✓**	**✓**	**✓**	**✓**	**✓**		**✓**	**✓**	**✓**	**✓**					
[40]	**✓**	**✓**	**✓**		**✓**	**✓**	**✓**	**✓**								**✓**
[44]		**✓**	**✓**			**✓**			**✓**	**✓**	**✓**	**✓**	**✓**			**✓**
[59]						**✓**			**✓**	**✓**	**✓**					**✓**
[45]		**✓**		**✓**					**✓**	**✓**		**✓**	**✓**			**✓**
[57]		**✓**	**✓**			**✓**	**✓**	**✓**	**✓**							
[51]	**✓**	**✓**	**✓**								**✓**	**✓**	**✓**			
[52]		**✓**	**✓**			**✓**	**✓**	**✓**	**✓**							
[42]						**✓**				**✓**						
[46]						**✓**			**✓**		**✓**					
[56]		**✓**	**✓**			**✓**	**✓**	**✓**								**✓**
[41]	**✓**	**✓**	**✓**			**✓**	**✓**	**✓**	**✓**							
[43]		**✓**	**✓**			**✓**	**✓**	**✓**	**✓**							
[60]	**✓**	**✓**	**✓**													**✓**
[50]		**✓**	**✓**		**✓**			**✓**	**✓**		**✓**	**✓**				**✓**
[49]		**✓**	**✓**		**✓**				**✓**		**✓**	**✓**				**✓**
[58]		**✓**	**✓**				**✓**	**✓**			**✓**					

**Table 6:** Sub-fields of applications based on medical specialties in PI preventions.

Area		Reference	Author(s)	Year	Country	Type	Dataset Size	Algorithms	Input of the Model	Output of the Model
**Posture bed** **recognition**	DL	[67]	Chiang et al.	2022	China	Journal	Seven different sets of samples that have been trained and tested; each set has its own number of images (Table 3 in [67])	CNN	3D skeleton of PI patients that has articulated joints	Skeleton-based posture classification of elderly patients with PI
	DL	[68]	Cicceri et al.	2020	Italy	Journal	N/A	DNN, SVM, RF	Data was collected through internal sensors that estimate the position of PI patients	Classify the position of PI patients based on sensors and send a notification to change the patient’s body position when a patient remains in the same position for a while
	DL	[69]	Heydarzadeh et al.	2016	US	Conference	60,000 PI images	Deep autoencoder neural network- Histogram of Gradient (HoG), PCA-SVM, Bayesian inference, Kurtosis-Skewness, Gaussian Mixture Model (GMM)	A commercial pressure mapping system was used to gather the data	Classify in-bed into posture: right foetus, right yearner, supine, left yearner, and left foetus
	ML	[70]	Matar et al.	2020	Canada	Journal	1728 sensors	MLP	Bed-sheet pressure sensors were used to collect the signals of body pressure/ pressure image	Autonomous approach to classify bed posture: spine, right, left, and prone
	ML	[71]	Duvall et al.	2019	US	Journal	4 sensors	KNN	Data were collected through e-scale positioned under the bed to measure the weights of each leg on the bed	Classify types of movement in bed: turn in place, roll, extremity movements, and assisted turn
	ML	[72]	Enayati et al.	2018	US	Conference	4 sensors	PCA, NN	Pressure sensors were used to collect signals of body pressure from 58 patients	Classify the most common four sleeping postures of the patients: left lateral, right lateral, supine, and prone
	ML	[73]	X. Xu et al.	2016	China	Journal	a 3 × 3 pressure sensor array	Developed skew-based sleep posture classifier based on KNN	Pressure sensors were used to collect signals of body pressure/pressure image	Predict sleep posture recognition based on Body-Earth Mover’s Distance (BEMD)
	ML	[74]	Baran Pouyan et al.	2016	US	Journal	1728 sensors	KNN	Commercial pressure map model, which has sensors, were used to capture pressure data continuously/ pressure image	Clustering model to extract body limbs from pressure data gathered by a commercial pressure map device
	ML	[75]	Hsiao et al.	2015	China	Journal	5 Force Sensing Resistor (FSR)	Fuzzy theory, KNN, SVM	A pressure sensing pad was developed and used to collect signals of body pressure/pressure image	Classify the position of the PI patients in nursing homes, then send a notification when a patient remains in the same position for a while to change the patient’s body position
	ML	[76]	Pouyan et al.	2014	US	Conference	2048 pressure sensors, the model was tested on 15 different patients	KNN, NB, DT	Pressure image of bed inclination	Classify the most common three-bed inclination: B0 degree, B30 degree, and B60 degree
	ML	[77]	Barsocchi	2013	Italy	Conference	3 sensors	SVM, KNN	Receive signals of the body through signal strength, and transmit signals from a wireless appliance to a server	Classify the position of the PI elderly patients based on sensors, and observe the activities of patients who cannot move their bodies the way they should
**Posture wheel-chair** **recognition**	ML	[78]	Jaffery et al.	2022	Saudi Arabia	Journal	Matrix configuration (9 sensors), and cross configuration (5 sensors)	KNN, LR, DT, SVM, LightGBM	Capture a real-time posture/signals of a patient on a wheelchair seat using sensors (two configuration systems)	Recognize the sitting posture of wheelchair users to prevent PI. The five positions are ideal, left-leaning, forward-leaning, right-leaning, and backward-leaning
	ML	[79]	Ma et al.	2017	China	Journal	12 sensors	DT, SVM, MLP, NB, KNN	Collect the posture of patients through sensor configuration	Capture cushion-of posture of wheelchair
**Biomedical modeling (i.e., ML** **model to learn the** **pattern between pressure map modes and strain field modes)**	ML	[80]	Grunerbel et al.	2022	Germany	Conference	A pressure sensor and a vital parameter sensor node: collect data for 17 nights	A multivariate subsequence clustering algorithm	Collect signals of skin temperature, SpO2, and heart rate	Measure skin temperature and blood oxygen saturation around potential wound sites in addition to pressure loads. Then send a medical alarm based on the status of the patients
	ML	[81]	Luboz et al.	2018	France	Journal	19 pressure modes	N/A	Collect signals of skin, fats, and muscles in real-time	Design a 3D buttock model to provide PI prevention (skin detection)

**Table 7:** Sub-fields of applications based on medical specialties in PI Assessment.

Area		Reference	Author(s)	Year	Country	Type	Dataset Size	Algorithms	Input of the Model	Output of the Model
**Systematic** **Review**	Approach	[112]	Kaswan et al.	2020	Malaysia	Journal	A brief review of wound classification and wound segmentation, most of the literature was imported from the dataset of national pressure ulcer advisory panel (10 studies)			
		[4]	Zahia et al.	2019	Spain	Journal	A systematic review was conducted by analyzing 114 studies related to wound analysis in general, using image processing			
**Wound** **Segmentation**	DL	[83]	Ramachandram et al.	2022	Canada	Journal	58 images	CNN	Wound images of PI, arterial ulcers, and venous ulcers	Wound tissue segmentation
	DL	[84]	C. W. Chang et al.	2022	China	Journal	2893 images	U-Net, DeeplabV3, PsPNet, FPN, and Mask R-CNN	Wound images of PI	Wound tissue segmentation
	ML	[85]	Howell et al.	2021	US	Journal	199 images	Droice Labs wound analytics service	Wound images of PI	Wound area and granulation tissue tracing/wound boundary detection
	DL	[86]	C. Wang et al.	2020	US	Journal	1109 images	CNN based on MobileNetV2	PI foot ulcer images	Segment wound regions/boundary detection
	DL	[87]	Ohura et al.	2019	Japan	Journal	440 images	CNN and TL	Wound detection/ boundary detection	Wound detection/ boundary detection
	DL	[3]	Zahia et al.	2018	Spain	Journal	22 high-resolution images per class, and then cut into 5*5 sub- images, ending with 380,000 small images	CNN	PI wound images	Tissue segmentation: granulation, slough, and necrotic tissues
	DL	[88]	García-Zapirain et al.	2018	Spain	Journal	193 images	3D CNN	PI wound images	Tissue segmentation: granulation, slough, and necrotic tissues
	ML	[89]	Garcia-Zapirain et al.	2017	Spain	Journal	48 images	Developed their own framework	PI wound images	Design a segmentation software for image segmentation and wound detection
	ML	[90]	F. J. Veredas et al.	2015	Spain	Journal	113 images	k-means, NN, SVM, RF	Wound images of PI patients with home-care assistance	Tissue identification, then classify wound tissue types: necrosis, slough, granulation, and healing skin
	ML	[92]	F. Veredas et al.	2010	Spain	Journal	113 images	PCA, MLP, NB	PI wound images	Tissue identification in wound image/ recognition for different types of tissue: necrosis, slough, granulation, healing, skin, and global
	ML	[91]	Wannous et al.	2007	France	Conference	905 images/regions (granulation: 302, slough: 243, necrosis: 73, and healthy: 287)	SVM	PI wound images	Segment wound regions/boundary detection (granulation, slough, and necrosis)
**Wound** **Classification**	DL	[93]	Ay et al.	2022	Turkey	Journal	1091 images	Deep TL, CNN	PI wound images	Classification of four stages of PI: stages 1–4
	ML	[94]	Fergus et al.	2022	UK	Conference	216 images	NN	PI wound images	Classification of six stages of PI: stage 1–4, unstageable PI, and DTPI
	DL	[95]	Liu et al.	2022	China	Journal	327 images	ResNet-v2 model (CNN)	PI wound images	Wound classification in two phases: phase 1: erythema or non- erythema; phase 2: “extensive necrosis or moderate necrosis
	DL	[96]	Anisuzzaman et al.	2021	US	Conference	2176 images collected from three datasets (730, 358, 1088)	TL and MMML	Wound images and corresponding locations	Wound classification: diabetic, pressure, venous ulcers, and surgical
	DL	[97]	Matsumoto et al.	2021	Japan	Journal	860 images	CNN	Ultrasound images of PI	Classification of types of DTPI: unclear layer structure, cobblestone-like pattern, cloud-like pattern, and anechoic pattern
	DL	[98]	A. Yilmaz et al.	2021	Turkey	Conference	175 images	CNN	PI wound images	Classification of six stages of PI: stage 1–4, unstageable PI, and DTPI
	ML	[99]	B. Yilmaz et al.	2021	Turkey	Conference	142 images	LR, NN	PI wound images	Classification of six stages of PI: stage 1–4, unstageable PI, and DTPI
	ML	[100]	Mombini et al.	2021	US	Conference	2056 images	XGboost, DT, RF, SVM	PI chronic images	Classifying the status of the wound into maintaining current treatment, referring the patient to a specialist, changing the current treatment
	DL	[101]	D. H. Chang et al.	2021	China	Conference	210 images	U-net CNN	PI wound images	Tissue classification and severity evaluation of wound condition and severity: granulation > 90%, granulation 70–90, granulation < 30, necrosis < 50, necrosis > 50
	ML	[102]	Kavitha et al.	2017	US	Conference	59 images	MLP, SVM, RF, NB	wound images (leg ulcers, venous and arterial, and pressure)	Classify images into pressure ulcer vs. leg ulcers
**Wound** **Healing**	ML	[103]	Lustig et al.	2022	Israel	Journal	173 images	Developed their own algorithm	Subepidermal moisture delta	Predict heel deep tissue injuries for ICU patients (heal or not within seven days)
	ML	[104]	Chun et al.	2021	China	Journal	152 images	RF, XGBoost	EHR variables	Classifying pediatric patients into healing or delayed healing
	ML	[105]	F. J. Veredas et al.	2010	Spain	Conference	743 images	SVM, NB, NN, DT	PI wound images (sacrum and hip)	Predict the wound status: improvement or improvement delayed
**Wound** **Measurement**	ML	[106]	Silva and Machado	2021	Brazil	Journal	105 images	SVM-Grabcut	PI wound images	Measurement of the “area” affected by PI
	ML	[107]	D. Li and Mathews	2017	US	Journal	32 images	Developed their own model using SVM and Gaussian model	3D wound images	Measure size of PI
**Wound** **Segmentation and Wound** **Measurement**	DL	[108]	Zahia et al.	2020	Spain	Journal	210 images	Mask RCNN	3D mesh and 2D wound images	Measurement of depth, area, volume, major axis, and minor axis (external segmentation of the wound)
	DL	[109]	Chino et al.	2020	Brazil	Journal	446 images	CNN	Wound images (two datasets)	Wound tissue segmentation, and measurement size of PI
	ML	[110]	F. J. Veredas et al.	2009	Spain	Conference	50 images	A hybrid model: NN and Bayesian classifiers	Wound images of PI	Wound segmentation into wound regions (separate wounds from healing areas) then measure area of wounds
**Wound** **Segmentation, Classification,** **Measurement, and Healing**	ML	[111]	M. C. Chang et al.	2018	US	Journal	133 scanning sessions from 23 enrolled subjects	Developed their own algorithm	Multimodal PI images: 3D depth, RGB, chemical sensing, thermal, and multispectral	Tissue classification (granulation vs. slough), 3D depth: 3D wound size measurement (length, width, depth, surface, and volume), thermal profiling, and chemical sensing: heal trend analysis

## Data Availability

Not applicable.
